# Case Report: Novel compound heterozygous mutations in *PNPLA6* gene associated with Oliver-McFarlane syndrome

**DOI:** 10.3389/fgene.2025.1660243

**Published:** 2025-11-06

**Authors:** Jia Zheng, Zhe Wang, Keqing Li, Lixia Chen, Yayin Luo, Fei Yu, Dan Wang, Guangxiang Yu

**Affiliations:** 1 Department of Neurology, The First Affiliated Hospital of Dalian Medical University, Dalian Medical University, Dalian, China; 2 Department of Neurology, Anshan Central Hospital, Anshan, China

**Keywords:** Oliver-McFarlane syndrome, patatin-like phospholipase domain containing 6, whole exome sequencing, variants, clinical characteristics

## Abstract

**Background:**

Oliver-McFarlane syndrome (OMCS) is an extremely rare congenital disorder that presents with hypogonadotropic hypogonadism, long eyelashes and eyebrows, pigmentary retinopathy, peripheral nerve axon neuropathy and other associated features. It is currently known that OMCS is linked to variants in the patatin-like phospholipase domain containing 6 (*PNPLA6*) gene, but the specific pathogenic mechanism is still unclear.

**Methods:**

We performed Whole exome sequencing (WES) on the proband and his parents, followed by validation of the findings through Sanger sequencing and Reverse Transcription-Polymerase Chain Reaction (RT-PCR) analysis.

**Results:**

Sanger sequencing identified two compound heterozygous variants in the *PNPLA6* (NM_006702.5) gene in the proband: c.3184G>A (p.Val1062Met) and c.2704-18C>G. According to the ACMG guidelines, the c.3184G>A variant is classified as likely pathogenic, while the c.2704-18C>G variant is discovered for the first time. Segregation analysis further revealed that the c.3184G>A variant was inherited from the father, whereas the c.2704-18C>G variant was derived from the mother—consistent with an autosomal recessive inheritance pattern. RT-PCR detected that the c.2704-18C>G variant caused a 29bp deletion upstream of exon 26, resulting in a splice site mutation (p.His902Alafs108).

**Conclusion:**

We report a case from China of *PNPLA6* gene variants leading to Oliver-McFarlane syndrome, with the patient exhibiting typical characteristics of OMCS. Our findings further substantiate the pathogenicity of *PNPLA6* gene variation in OMCS, broadening the established genotypic spectrum of the *PNPLA6* gene. These findings enhance the understanding of its pathogenesis and offer perspectives for clinical diagnosis and management.

## 1 Introduction

Oliver-McFarlane syndrome (OMCS) is a rare congenital disorder first reported in 1965 by Oliver and McFarlane (Oliver, 1965). OMCS is characterized by short stature, genital dysplasia, long eyelashes, long eyebrows, severe chorioretinal atrophy and deficiency of various pituitary hormones, including growth hormone, gonadotropins and thyroid-stimulating hormone (Lisbjerg et al., 2021). This disease is currently known to be exclusively associated with variants in the patatin-like phospholipase domain containing 6 (*PNPLA6* gene) (Hufnagel et al., 2015).

The *PNPLA6* gene is located on chromosome 19p13.2, composed of 34 exons, and encodes neuropathy target esterase (NTE), a highly conserved phospholipase. This enzyme deacetylates intracellular phosphatidylcholine, producing glycerophosphocholine, and is believed to play a role in the growth of neural processes and axonal elongation during neuronal differentiation (Tang et al., 2019). Mutations in the *PNPLA6* gene can disrupt the enzymatic activity of *PNPLA6*, impairing synaptic connections in neuronal networks, leading to a range of diseases from intellectual disabilities to ataxia. Mutations in this gene have been confirmed to be associated with specific syndromes, including Boucher-Neuhauser syndrome (BNHS), Laurence-Moon syndrome (LNMS), Oliver-McFarlane syndrome and autosomal recessive hereditary spastic paraplegia type 39 (SPG39) (Synofzik et al., 1993). Here, we report a case of Oliver-McFarlane syndrome caused by *PNPLA6* gene mutations from China.

## 2 Materials and methods

### 2.1 Patient consents and standard protocol approvals

Genomic DNA was extracted from peripheral blood from the patient and his healthy parents. Written informed consent was obtained from the patient’s parents. The present study was ethically approved by the Ethics Committee of The First Affiliated Hospital of Dalian Medical University.

### 2.2 Exome sequencing and data analysis

Whole exome sequencing (WES) was performed using the IDT xGen Exome Research Panel v2.0. High-throughput sequencing was performed by BGI DNBSEQ-T7 (PE150). The sequencing process was performed by Beijing Chigene Translational Medicine Research Center Co. Ltd. The reliability of the whole exome sequencing data was confirmed by the following quality control metrics: the average sequencing depth ≥100×, and the 20× coverage rate of the target region ≥95%, Q20 percentage (indicating a ≤1% error probability) ≥95%, Q30 percentage (indicating a ≤0.1% error probability) ≥85% and GC content 48%–51%.

Raw Data Preprocessing: Adapters and low-quality reads were trimmed using fastp. Read Alignment: Clean reads were aligned to the Ensembl GRCh37/hg19 reference genome with the Burrows-Wheeler Aligner (BWA). Variant Calling & Filtering: Single nucleotide polymorphisms (SNPs) and short insertions/deletions (indels) were called using the Genome Analysis Toolkit (GATK). High-confidence variants were filtered with criteria: minimum sequencing depth (DP) ≥5×, minimum genotype quality (GQ) ≥35, and allele balance (for heterozygous variants) between 10% and 85%. Minor Allele Frequency (MAF) Annotation: Variants were annotated for MAFs using Chigene’s in-house online system (www.chigene.org), leveraging databases such as the 1000 Genomes Project, dbSNP, ESP, ExAC, and Chigene in-house MAFs database. Protein Structural Impact Prediction: Functional alterations in protein structure were predicted using PROVEAN, SIFT, PolyPhen-2 (HDIV/HVAR), MutationTaster, M-Cap, and REVEL. Splice Site Functional Impact Prediction: Effects on splice sites were assessed via MaxEntScan, dbscSNV, and GTAG. Pathogenicity Annotation: Variants were evaluated for pathogenicity following the ACMG guidelines, with annotations cross-referenced against OMIM, HGMD, and ClinVar databases. Co-segregation & Phenotype Correlation Analysis: Finally, co-segregation analysis (to validate inheritance patterns) and phenotype correlation analysis (to link genetic variants with clinical manifestations) were conducted.

### 2.3 Sanger sequencing

Sanger sequencing was employed to validate the presence of the c.3184G>A and c.2704-18C>G mutations in the proband and his parents. The primers used for PCR amplification are detailed in Supplementary Table S1. The PCR amplicons were subjected to Sanger sequencing on an ABI 3730XL sequencer, followed by sequence analysis utilizing DNASTAR software and alignment against the reference sequence of the *PNPLA6* gene (NM_006702.5).

### 2.4 Reverse transcription sequencing (cDNA sequencing)

This experiment was designed to verify whether the c.2704-18C>G variant of the *PNPLA6* gene (a variant near the splice site of intron 25) disrupts normal mRNA splicing, detect the presence of aberrant transcripts in the patient (e.g., exon skipping, intron retention), and further clarify the impact of this variant on gene function (e.g., whether it causes frameshift or protein truncation). Total RNA was extracted from both patient and control samples using the Beyo Tech Blood RNA Mini Extraction Kit, followed by reverse transcription into cDNA. Specific RT-PCR amplification was performed simultaneously on patient and control cDNA samples targeting the *PNPLA6* gene (888 bp product) and the reference gene *WDR*45 (508 bp product). The primers used for RT-PCR amplification are detailed in Supplementary Table S2.

## 3 Results

### 3.1 Case clinical description

The patient is a 31-year-old young male with the following clinical features: height 165 cm, weight 52.5 kg, absence of an Adam’s apple, thick eyebrows and eyelashes, sparse facial and axillary hair, genital Tanner stage I, pubic hair Tanner stage III-IV and pes cavus. The patient was born full-term via vaginal delivery, but exhibited slow growth. At the age of 9, he experienced bilateral visual decline and was suspected to exist growth hormone deficiency. He received growth hormone therapy for 2–3 months but discontinued treatment on his own, with no significant improvement in vision. At 21 years old, he sustained an ankle fracture in a car accident and after orthopedic treatment, he still had gait instability. By the age of 31, he developed involuntary head tremors, which worsened with emotional stress or postural changes. The patient has been treated sequentially with Almarl 5 mg twice daily to reduce involuntary tremors and Idebenone 30 mg three times daily to improve cerebral metabolism and alleviate psychiatric symptoms, both of which have been confirmed to be effective. There is no family history of similar conditions in his extended relatives.

**FIGURE 1:**
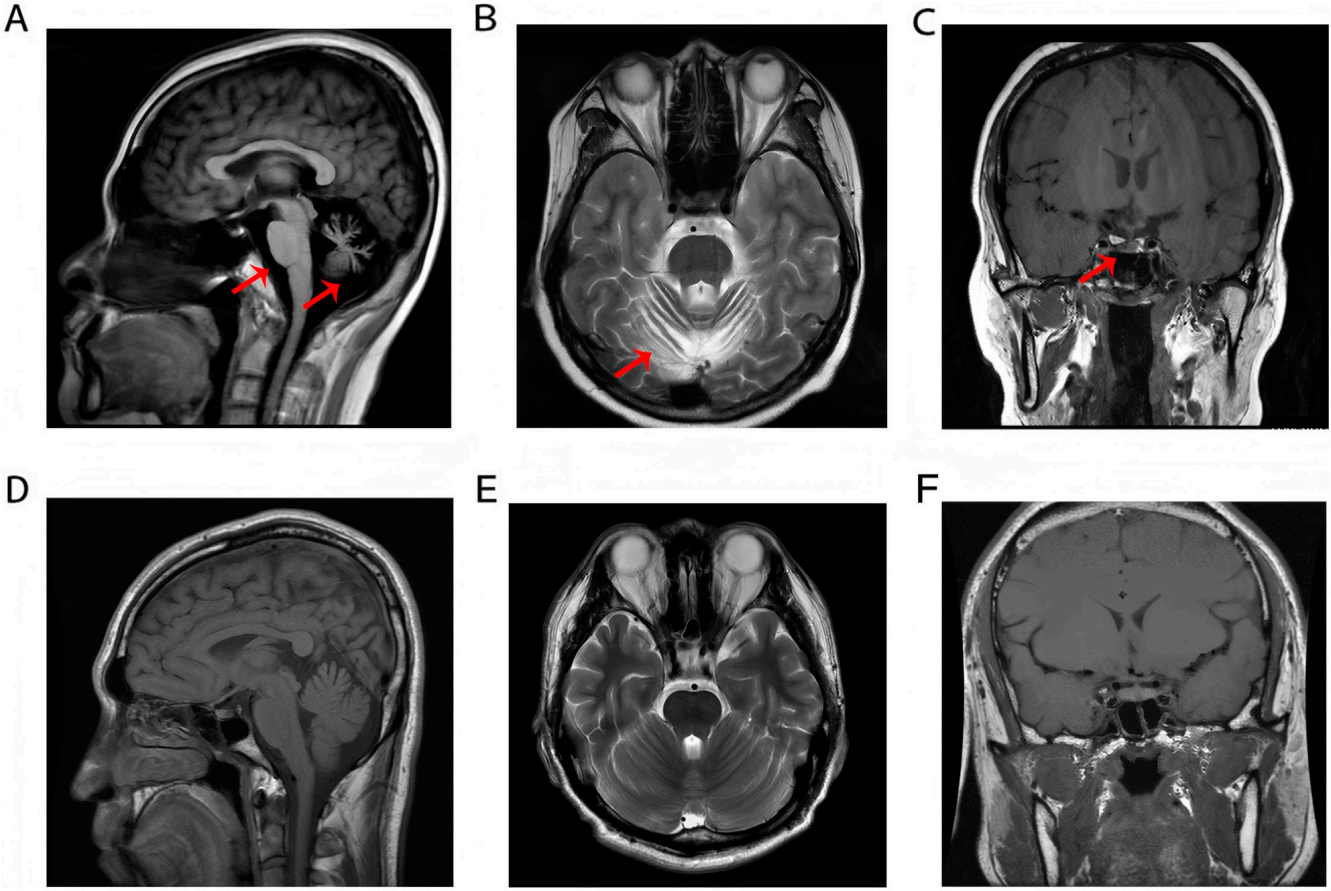
**(A–C)** Cerebellar atrophy with brainstem thinning and anterior pituitary atrophy. **(D–F)** Normal MRI demonstrates preserved anatomical morphology and homogeneous signal intensity of the pons, medulla oblongata, cerebellum, and pituitary gland in a healthy control subject.

Brain Magnetic Resonance Imaging (MRI) revealed cerebellar atrophy with mild thinning of the pons and medulla oblongata (Figures 1A,B). Coronal MRI reveals atrophy of the anterior pituitary lobe, revealing diffuse volume loss and symmetric thinning to a height of 1–2 mm, and an anatomically normal posterior pituitary and stalk (Figure 1C). These features were compared with normal control MRIs (Figures 1D–F). Fundoscopic examination revealed pale optic discs with blurred margins, tortuous retinal arteries and veins, extensive atrophy of the posterior pole, and peripheral retinal depigmentation with pigmentary changes. The macular foveal reflex was absent. Optical coherence tomography (OCT) of both eyes demonstrated thinning and atrophy of the macular retina (Supplementary Figure S1). The hormone profile of the pituitary-thyroid axis revealed a markedly suppressed thyroid-stimulating hormone (TSH) level of <0.005  mIU/L, elevated free triiodothyronine (FT3) at 11.37 pmol/L, elevated free thyroxine (FT4) at 36.25 pmol/L, and a thyrotropin receptor antibody (TRAb) level of 5.97 IU/L. These findings collectively indicated thyrotoxicosis. Hormonal assessment of the pituitary-gonadal axis revealed decreased levels of testosterone (TES), luteinizing hormone (LH), follicle-stimulating hormone (FSH), and androstenedione (AND) establishing the diagnosis of hypogonadotropic hypogonadism.

### 3.2 Compound heterozygous variants of the *PNPLA6* gene were found in the OMCS patient

**FIGURE 2:**
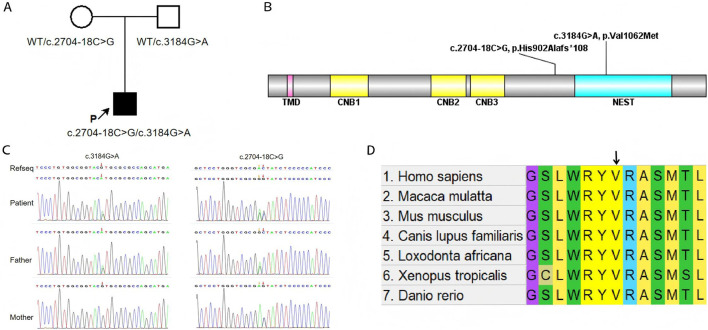
**(A)** The schematic diagram to illustrate the inheritance pattern of the identified variants. **(B)** Locations of variants from patient relative to functional domains in *PNPLA6*. Abbreviations of domains: TMD, transmembrane domain; CNB, cyclic nucleotide binding domains; NEST, Neuropathy target esterase domain. **(C)** Compound heterozygous *PNPLA6* variants in the proband: c.3184G>A (p.Val1062Met, paternal) and c.2704–18C>G (maternal). The red arrow indicates the mutation site. **(D)** Conservative prediction: the results from comparing protein sequences across multiple species show that this site is highly conserved.

**FIGURE 3:**
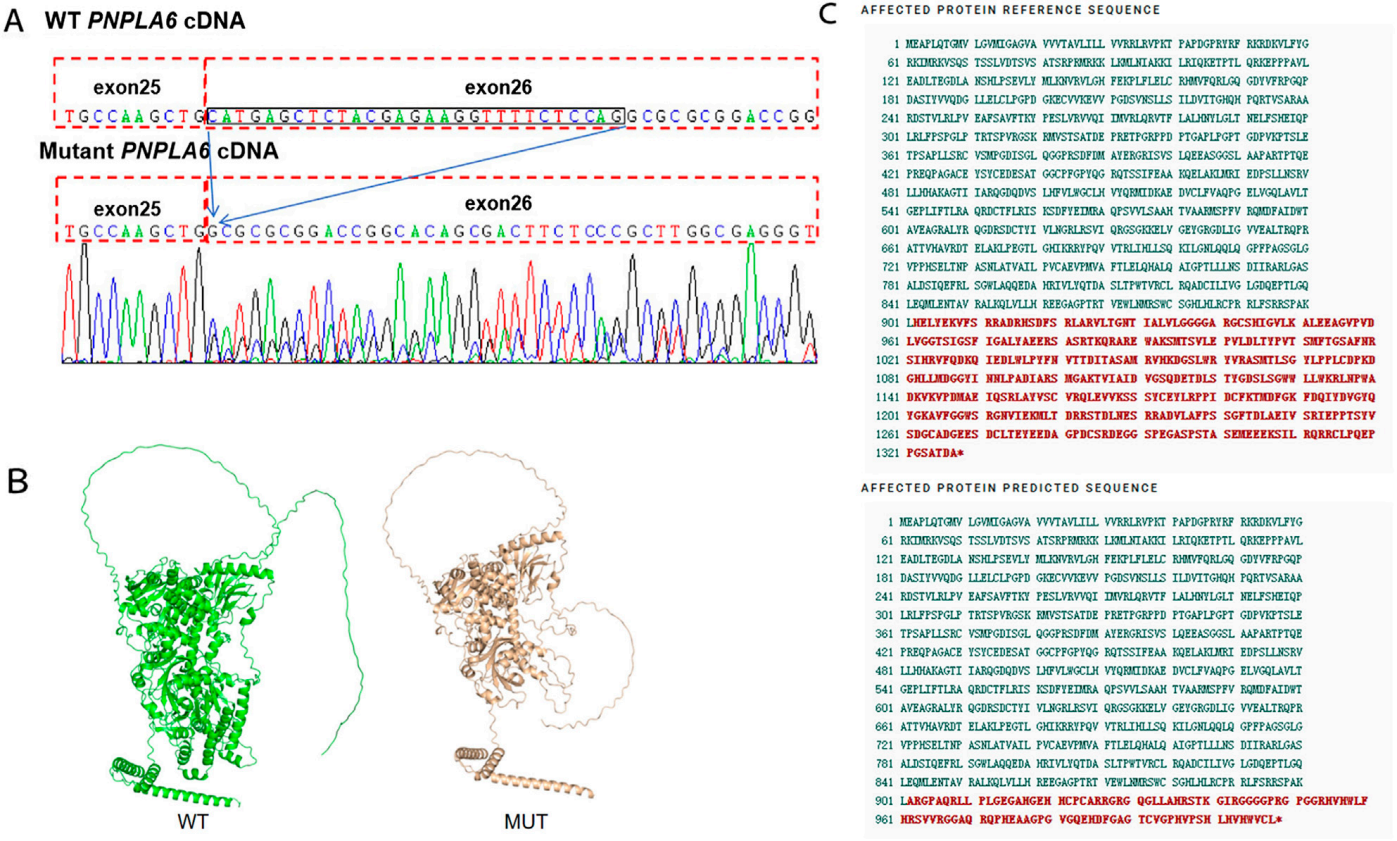
**(A)** Variant marker site of the *PNPLA6* gene c.2704–18C>G. **(B)** The AlphaFold-predicted model of the aberrant protein structure. WT: Wild Type, MUT: Mutant. **(C)** The c.2704-18C>G variant causes premature termination of protein synthesis.

To further investigate the genetic basis of the patient’s condition, WES was performed. The analysis identified two heterozygous variants in the *PNPLA6* (NM_006702.5) gene: c.3184G>A (p.Val1062Met) and c.2704–18C>G (Figures 2A,B). Segregation analysis further revealed that the c.3184G>A variant was inherited from the father, whereas the c.2704–18C>G variant was derived from the mother (Figure 2C). The first variant, c.3184G>A, results in a nucleotide substitution from guanine (G) to adenine (A) at position 3,184, leading to an amino acid change from valine (Val) to methionine (Met) at position 1,062 (a missense mutation). This variant has been documented in the dbSNP database under the accession number rs587777182. According to the ACMG classification framework, this variant was classified as likely pathogenic (PM1+PM2_Supporting + PM3+PP2+PP3) (Table 1). Conservation analysis was performed using MEGA X software (http://www.megasoftware.net), which revealed that this site is highly conserved across multiple species (Figure 2D). Pathogenicity prediction tools, including Provean, SIFT, Polyphen2_HDIV, Polyphen2_HVAR and MutationTaster, consistently indicated that this variant is likely to affect protein function. The substitution of Val with Met disrupts the stability of the α-helical scaffold, causing structural disorganization of the catalytic funnel and misalignment of the Ser1014/Asp1144 dyad, which significantly impairs or abolishes the esterase activity of *PNPLA6* (Synofzik et al., 2014). The second variant, c.2704–18C>G, is located within intron 25 and involves a substitution of cytosine (C) to guanine (G) at the −18 site of nucleotide 2,704 of the *PNPLA6* coding sequence representing a splice site-proximal intronic mutation. Splicing prediction tools, such as MaxEntScan and GTAG suggested that this variant may disrupt normal mRNA splicing. Sanger sequencing of the proband’s sample illustrates wild-type (WT) and mutant *PNPLA6* cDNA spanning exons 25–26, demonstrating the aberrant splicing at this locus. Specifically, the mutant trace reveals a heterozygous 29-bp deletion upstream of exon 26 (marked by the red bracket), which disrupts the canonical splice junction between exons 25 and 26—in contrast to the WT trace, which exhibits seamless splicing of exons 25 and 26 with no deletions. These sequencing data confirm the presence of abnormal splicing (Figure 3A). The predicted amino acid alteration was identified as NM_006702.5:c.2704_2732del,p. (His902Alafs*108). This variant results a 29-nucleotide deletion (c.2704_2732del) in the coding region, causing a frameshift mutation. The original histidine at position 902 is replaced by alanine, followed by premature termination of protein synthesis after 108 amino acids downstream due to encounter with a stop codon (Figures 3B,C). Such a frameshift alteration is likely to severely impact protein structure and function. According to the ACMG classification framework, this variant was classified as likely pathogenic (PM2_Supporting + PM3+PP3+PS3 _Moderate).

**TABLE 1:** Summary of silico pathogenicity predictions and ACMG rating for the c.3184G>A (p.Val1062Met) variant.

Prediction tool/classification	c.3184G>A (p.Val1062Met) prediction result
Protein domain	NEST
Provean	Deleterious (-2.62)
SIFT	Damaging (0.001)
Polyphen2_HDIV	Probably damaging (1.0)
Polyphen2_HVAR	Probably damaging (0.98)
MutationTaster	Disease_causing (0.999957)
M-CAP	Damaging (0.432295)
REVEL	Deleterious (0.818)
CADD	Deleterious (26.5)
metaRNN	Deleterious (“D”)
ACMG	Likely pathogenic, PM1+PM2_Supporting + PM3+PP2+PP3

## 4 Discussion

In this case report, we identified a rare case of Oliver-McFarlane syndrome which exhibited the classic clinical features of OMCS, including coarse hair, retinal degeneration and atrophy, hypogonadotropic hypogonadism, reproductive dysfunction (Shi et al., 2023), and manifestations of spinal cord and cerebellar involvement (Liu and Hufnagel, 2023). The disease is currently known to be associated exclusively with variants in the *PNPLA6* gene (Hufnagel et al., 2015). Extensive *in vivo* and *in vitro* studies have been conducted to elucidate the pathogenic mechanisms of the *PNPLA6* gene mutations which is known to play a crucial role in the development of the retina, choroid, pituitary gland, cerebellum and ventricles (Hufnagel et al., 2015). The *PNPLA6* gene encodes neuropathy target esterase (NTE) (Pujar et al., 2018; Liu et al., 2024). NTE is a phospholipase located in the endoplasmic reticulum. NTE plays a pivotal role by deacylating through two pathways involving phosphatidylcholine and lysolipid phosphatidylcholine (Deik et al., 2014; Chang et al., 2019). Firstly, NTE can deacylate phosphatidylcholine, a major component of biological membranes, into its constituent fatty acids and glycerophosphocholine (Read et al., 2009). Glycerophosphocholine serves as a precursor for the biosynthesis of acetylcholine, a key neurotransmitter involved in mediating cell signaling within the nervous system. This disruption may lead to impaired development and maintenance of synaptic connections across various neuronal networks. Secondly, NTE can also significantly hydrolyze certain lysolipids, with lysolipids making up 3%–4% of total phospholipids in the Golgi membrane suggesting that it may lead to structural changes in the Golgi apparatus, playing a role in membrane transport and protein secretion modulation (Van Tienhoven et al., 2002). Given the role of NTE in membrane transport, Topaloglu et al. found through studies on LβT2 cells (a cell line that inhibits NTE activity) that NTE does not directly participate in GnRHR signaling. Instead, it reduces the response of luteinizing hormone to gonadotropin-releasing hormone by decreasing the exocytosis of LH stimulated by GnRH leading to neurodegeneration and impaired pituitary gonadotropin release, resulting in hypogonadotropic hypogonadism (Topaloglu et al., 2014). Studies by Moser (Moser et al., 2004) and Pamies (Pamies et al., 2010) on mouse embryos have demonstrated that the *PNPLA6* gene is essential for placental formation, development of extraembryonic yolk sac vasculature and embryonic viability. Mouse embryos with silenced *PNPLA6* expression were nonviable. Pamies et al. conducted the first study on human-derived NTera2/D1 (hNT2) embryonal carcinoma stem cells, confirming that the *PNPLA6* gene plays a role in the early stages of neuro differentiation *in vitro*, with its primary biological impacting neurogenesis and epithelial tube morphogenesis (Pamies et al., 2014). Numerous *in vivo* and *in vitro* studies have confirmed that *PNPLA6* affects multiple nervous systems, from the retina to the cerebellum, upper and lower motor neurons, and the neuroendocrine system. Dysfunction of this protein can lead to a wide spectrum of neurodegenerative disorders with overlapping clinical features (Pamies et al., 2014).

Previous studies have shown that most variants in the *PNPLA6* gene cluster within the 5,100 residues of the phospholipid esterase domain (Synofzik et al., 2014). To date, 161 variants of the *PNPLA6* gene have been reported in Leiden Open Variation Database (Fokkema et al., 2021). We acknowledge that a comprehensive summary of the *PNPLA6* gene mutational spectrum along with the type and location of the mutation, has been previously established (Lisbjerg et al., 2021). The c.2704–18C>G variant is reported here for the first time, and is located near the splice site. For patients diagnosed with OMCS, pathogenic variants of *PNPLA6* have been confirmed, while pathogenic variants in other genes have not yet been reported. In this study, two heterozygous variants were identified in the *PNPLA6* gene. One variant, c.3184G>A is already documented in the dbSNP database as a missense variant which alters the amino acid at position 1,062 from valine to methionine. The *PNPLA6* p.Val1062Met variant localizes to one of three adjacent α-helices in the EST domain (the catalytic core of *PNPLA6*), which acts as a structural scaffold for maintaining the integrity of the domain’s funnel-shaped catalytic center and is close to the active site Ser1014/Asp1144 catalytic dyad. The substitution of Val with Met disrupts the stability of the α-helical scaffold, causing structural disorganization of the catalytic funnel and misalignment of the Ser1014/Asp1144 dyad, which significantly impairs or abolishes the esterase activity of *PNPLA6* (Synofzik et al., 2014). According to the ACMG guidelines, this variant is classified as likely pathogenic (Richards et al., 2015). It could substitute the nucleotide at the −18 site of nucleotide 2,704 of the *PNPLA6* coding sequence with a cytosine nucleotide replaced by a guanine nucleotide, which may result in abnormal cDNA splicing and leading to frameshift mutations in protein translation, causing premature termination of protein synthesis. We propose that the c.2704–18C>G variant may also be pathogenic. Our study detected two heterozygous variants: a missense mutation and a splice site mutation. By querying the Prosite database for information on the protein domains associated with these two variants, we found that the variant c.3184G>A (p.Val1062Met) is located within the patatin-like phospholipase domain, while c.2704–18C>G is located outside the domain. Research by Wu et al. suggested that the location and type of gene variants influence the phenotype and speculated that there could be race-specific alleles (Wu et al., 2021). He found that one allele or biallelic variant in the patatin-like phospholipase domain is highly associated with choroideremia and more patients with severe harmful *PNPLA6* variants exhibit choroideremia (Wu et al., 2021). The variants in this study match the conclusions above, but further studies with a larger sample sizes are needed to confirm these observations.

The principal limitation of this study is the absence of functional validation. Specifically, the lack of direct *in vitro* experiments (such as cell transfection or NTE enzymatic activity assays) means that the pathogenicity assessment currently relies on predictive algorithms and indirect evidence rather than direct demonstration of protein dysfunction. Subsequent research should include such functional studies to unequivocally confirm the pathogenic effects of the identified variants.

Overall, we have identified a novel case of OMCS with compound heterozygous variations in the *PNPLA6* gene. By exploring the critical role of the *PNPLA6* gene across various systems, we further confirmed its pathogenic role in OMCS. Currently, the mechanisms underlying *PNPLA6*-related disorders remain incompletely understood, and no disease-modifying therapies have been identified. However, potential therapeutic strategies, such as gene therapy or NTE replacement therapy, could be considered to alleviate clinical symptoms. As an extremely rare autosomal recessive disorder, OMCS requires further research to better understand its pathogenesis and provide new insights for diagnosis and treatment.

## Data Availability

The original contributions presented in the study are included in the article, further inquiries can be directed to the corresponding author.
