# Supplementary material for: Case Report: Novel compound heterozygous mutations in PNPLA6 gene associated with Oliver-McFarlane syndrome
Source: Front Genet. 2025 Nov 6;16:1660243. doi: 10.3389/fgene.2025.1660243 (PMC12631215; doi:10.3389/fgene.2025.1660243)
Supplement: Supplementary file 1 [file Supplementaryfile1.docx]

Supplementary Material

1. **Supplementary Figures and Tables**

## Supplementary **Tables**

Table 1: Primer sequences for PCR amplification of the two compound heterozygous variants.

| **Heterozygous Variants** | **c.3184G>A** | **c.2704-18C＞G** |
| --- | --- | --- |
| Gene Sequence Accession Number | NM_006702.5 | NM_006702.5 |
| Forward Primer Sequence | GTGGACGGGTGCTACGTTAACAA | CGCCCTTAAGCAGCTAGTCC |
| Reverse Primer Sequence | GTGTGTGTGGTGCGGGAGGTG | CAACGCTCCGATGAAAGAGC |

Table 2: Primer sequences for RT-PCR amplification of the *PNPLA6* gene and the internal reference gene *WDR45*.

| **Gene name** | ***PNPLA6*** | ***WDR45*** |
| --- | --- | --- |
| Gene Sequence Accession Number | NM_006702.5 | NM_007075.4 |
| Forward Primer Sequence | CTCGGAACTCACCAACCCAG | CTCGTCTGCTCCATTCACGA |
| Forward Primer Annealing Position | chr19:7618786-7618805 (exon22) | chrX:48933367-48933386 (exon9) |
| Reverse Primer Sequence | CGGTTAAAGGCAGACCCAGT | CACGTCGAAAGCCTCTCTGT |
| Reverse Primer Annealing Position | chr19:7621399-7621418(exon28) | chrX: 48932495-48932514(exon12) |
| Amplicon Length | 888bp | 508bp |
| Annealing Temperature | 60 | 60 |

## Supplementary Figure


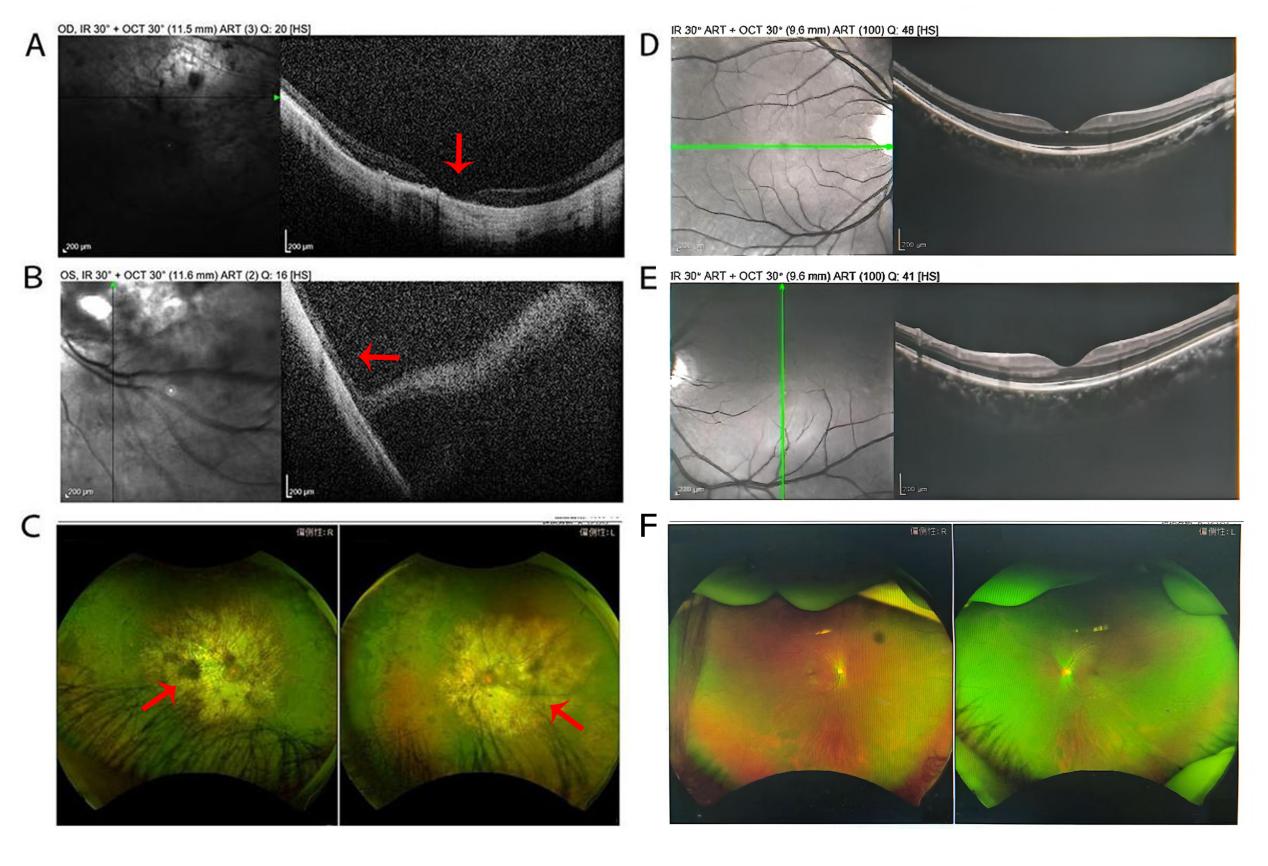


**Supplementary Figure 1.** (A, B, C) The optical coherence tomography demonstrates marked macular region retinal atrophy and thinning in the patient. (D, E, F) Representative optical coherence tomography from healthy control subjects shows physiological retinal architecture with well-defined layer stratification.
